# Antimony to Cure Visceral Leishmaniasis Unresponsive to Liposomal Amphotericin B

**DOI:** 10.1371/journal.pntd.0004304

**Published:** 2016-01-06

**Authors:** Gloria Morizot, Romain Jouffroy, Albert Faye, Paul Chabert, Katia Belhouari, Ruxandra Calin, Caroline Charlier, Patrick Miailhes, Jean-Yves Siriez, Oussama Mouri, Hélène Yera, Jacques Gilquin, Roland Tubiana, Fanny Lanternier, Marie-France Mamzer, Christophe Legendre, Dominique Peyramond, Eric Caumes, Olivier Lortholary, Pierre Buffet

**Affiliations:** 1 Plateforme ICAReB, Institut Pasteur, Paris, France; 2 Service d’anesthésie-réanimation, Hôpital Necker, Paris, France; 3 Service de Pédiatrie générale, Hôpital Robert Debré, Paris, France; 4 Service de Maladies Infectieuses et Tropicales, Hôpital de la Croix-Rousse, Lyon, France; 5 Service de Maladies Infectieuses et Tropicales, Hôpital Pitié-Salpêtrière, Université Pierre et Marie Curie, Paris, France; 6 Université Paris Descartes, Centre d’Infectiologie Necker-Pasteur, Hôpital Necker-Enfants malades, IHU Imagine, Paris, France; 7 Service d'Accueil des Urgences pédiatriques, Hôpital Robert Debré, Paris, France; 8 Service de Parasitologie-Mycologie, Hôpital Pitié-Salpêtrière, Paris, France; 9 Service de Parasitologie-Mycologie. Hôpital Cochin, Faculté de Médecine, Paris Descartes, Paris, France; 10 Service de transplantation rénale, Hôpital Necker, Paris, France; 11 945 INSERM, Université Paris 6, Paris, France; Universidade Federal de Minas Gerais, BRAZIL

## Abstract

We report on 4 patients (1 immunocompetent, 3 immunosuppressed) in whom visceral leishmaniasis had become unresponsive to (or had relapsed after) treatment with appropriate doses of liposomal amphotericin B. Under close follow-up, full courses of pentavalent antimony were administered without life-threatening adverse events and resulted in rapid and sustained clinical and parasitological cure.

## Author Summary

Visceral leishmaniasis causes fever, enlargement of internal organs like the liver and the spleen, and leads to death if no treatment is given. It is caused by a microbe called *Leishmania* and affects children and adults in warm and temperate regions of the world. Antimony in different forms has been used to treat Visceral Leishmaniasis for almost one century and is still in use in several countries despite the fact that it sometimes displays toxic effects, especially in patients with underlying health problems. Because it is better tolerated and at least as effective as antimony, liposomal amphotericin B is now used as the first treatment for Visceral Leishmaniasis in Southern Europe. We observed that a small proportion of patients—especially those with an impaired immune system—do not cure even after several courses of liposomal amphotericin B. In 4 such patients with “unresponsiveness” to liposomal amphotericin B, antimony provided a sustained cure without major side effects. We conclude that when multiple failures or relapses occur after treatment with liposomal amphotericin B, antimony is a reasonable, potentially life-saving treatment option. These observations also suggest that “unresponsiveness” to amphotericin B results from unusual resistance mechanisms.

## Introduction

Untreated visceral leishmaniasis (VL) is generally fatal. Although pentavalent antimony is still used in endemic areas [1], liposomal amphotericin B (L-AmB) has become the reference treatment in Europe and the US [2]. L-AmB is highly effective, generally better tolerated than pentavalent antimony, and requires fewer infusions. With L-AmB, treatment failure and VL relapse are rare in immunocompetent patients but frequent in immunocompromised patients [3]. Prolonged evolution has been observed despite L-AmB maintenance therapy [4–6], with poor prognosis, especially in HIV/HCV or HIV/HBV coinfected patients [7]. In such situations, high antileishmanial efficacy is required and severe adverse events are frequent. Miltefosine monotherapy is moderately effective in chronic uncontrolled VL [8]. High doses of pentamidine are toxic and paromomycin is not readily available in many countries [9]. We report the use of pentavalent antimony in 4 VL patients responding poorly to L-AmB.

## Materials and Methods

### Ethics Statement

Practitioners obtained oral non-opposition from duly informed patients to perform clinical data analysis.

**Fig 1:**
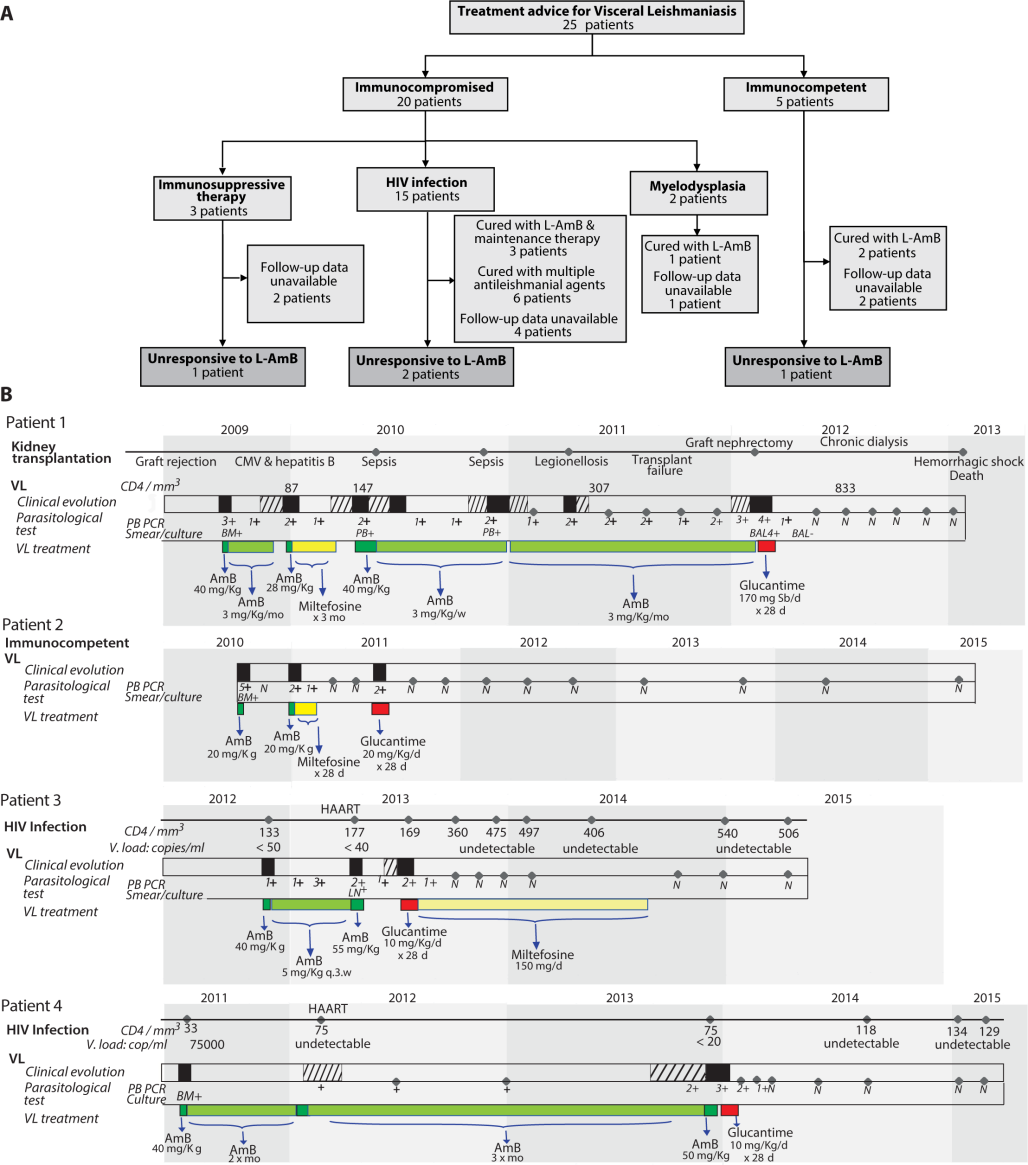
“black rectangle” VL episode; “hatched rectangle” oligosymptomatic VL; BM: bone marrow; PB: peripheral blood; LN: lymph node; BAL: broncho-alveolar lavage; q.3.w: every 3 weeks; mo: month; quantitative PCR (N = negative; 1+ = 0.5–10; 2+ = 11–100; 3+ = 101–1000; 4+ = 1001–100000; 5+ >100000 parasites/ml). **(A)Treatment advice for VL**: All patients received L-AmB as first-line treatment[2]; 4 became unresponsive to L-AmB as defined by failure or relapse with positive PCR for *Leishmania* after at least 2 complete courses (20–60 mg/kg cumulated dose/course). (B) Follow-up of 4 patients non-responders to L-AmB: Cure was defined as a negative smear, culture or PCR on leukoconcentrated blood plus remission of clinical signs and symptoms of VL with no relapse during 6 months. Relapse was defined as positive *Leishmania* PCR (any sample) associated with recurrence of ≥3 of the following markers: fever, splenomegaly, hepatomegaly, anemia, leukopenia, thrombocytopenia, weight loss, asthenia, digestive or cutaneous symptoms suggestive of VL. Patient information and consent were as per FNRCL procedures (http://www.parasitologie.univ-montp1.fr/conseil.htm). Doses of meglumine antimoniate (Glucantime) are expressed in mg of pentavalent antimony/kg.

From 2005 to 2013, treatment advice from a French National Reference Center for Leishmaniasis (FNRCL) expert was sought for 25 VL patients (Fig 1A). All received L-AmB as first-line treatment [2]; 4 became unresponsive (i.e., failure or relapse with positive *Leishmania* PCR after ≥2 complete courses (20–60 mg/kg cumulated dose/course, Fig 1B). Cure was defined as a negative smear, culture or PCR on leukoconcentrated blood plus remission of VL signs and symptoms with no relapse during 6 months. Relapse was defined as a positive *Leishmania* PCR in any sample associated with recurrence of ≥3 of the following markers: fever, splenomegaly, hepatomegaly, anemia, leukopenia, thrombocytopenia, weight loss, asthenia, digestive or cutaneous symptoms suggestive of VL. Previous explorations had shown that patients who respond to L-AmB have a several-Log decline in parasitemia (as assessed by quantitative PCR) in less than 3 weeks [10]. We used a kinetoplastic DNA target [11] using the following primers and probe: 15 pmol of direct primer (CTTTTCTGGTCCTCCGGGTAGG), 15 pmol of reverse primer (CCACCCGGCCCTATTTTACACCAA), and 50 pmol of TaqMan probe (FAM-TTTTCGCAGAACGCCCCTACCCGC-TAMRA).

### Patient 1

A 58-year-old male had a history of end-stage renal failure with 3 kidney transplantations (1979, 1981 and 2008). He had previous chronic hepatitis B, legionellosis, chronic Q fever and cytomegalovirus infection. The last renal transplantation was associated with acute rejection requiring a combination of anti-thymoglobulin, plasma exchanges, rituximab, mycophenolate mofetil 1g/d, tacrolimus 4.5mg/d and prednisone 10 mg/d. In 2004 *Leishmania* PCR in blood was positive (Fig 1B). L-AmB treatment initially induced clinical cure. Relapse occurred in August 2009 when plasma exchanges and corticosteroids boluses were administered for the graft rejection. Fever, anemia (Hb = 9.1g/dL) and leukopenia (leukocytes = 1600/mm^3^) were observed, along with 431 *Leishmania* per ml of blood (assessed by quantitative PCR). L-AmB (induction total dose, 40 mg/Kg) provided improvement and reduced parasitemia to 6.9 *Leishmania*/ml. A second recurrence with fever and a rise in parasitemia to 29.6 *Leishmania*/ml occurred in January 2010 during monthly maintenance administration of 3 mg/kg of L-AmB. High-dose L-AmB was reintroduced followed by miltefosine for 4 months. Parasitemia decreased but remained positive (0.4 *Leishmania*/ml) and number of CD4 lymphocytes increased from 87 to 147/mm3. A third recurrence was reported in July 2010 with fever, 29.8 *Leishmania*/ml and positive *Leishmania* culture from venous blood. L-AmB was reintroduced, followed by maintenance infusions for 4 months, 3 mg/Kg every 3 weeks plus downgrading of immunosuppressive therapies. As symptoms persisted, infusions were performed once-weekly and then twice-weekly. Parasitemia decreased from 88 to 0.6 *Leishmania*/ml but a fourth recurrence occurred in April-June 2011 with fever, leukopenia (leukocytes = 1200/mm3, CD4 lymphocytes = 186/mm3), anemia (Hb = 9.1g/dL) and parasitemia at 40 *Leishmania*/ml. Patient refused to increase the L-AmB dose, it continued at 3 mg/Kg twice-weekly. Transplant nephrectomy was performed in November 2011 and chronic dialysis reinstituted. In February 2012, fever, splenomegaly, anemia (Hb = 8.6g/dL) and thrombocytopenia (platelet count = 50,000/mm3) were observed with 12,870 *Leishmania*/ml and 60,440 *Leishmania*/ml of bronchoalveolar lavage fluid. Thereafter, meglumine antimoniate adapted to renal clearance was administered daily for 28 days, then 1–3 times/week for maintenance. Liver function remained normal. ECG showed a T-wave inversion but troponin remained normal. Fever and sweats resolved, spleen size decreased from 21 to 17 cm, and blood counts normalized in 4 weeks, number of CD4 lymphocytes = 833/mm3 in June 2012,. *Leishmania* blood PCR and cultures became negative within a month and remained negative over eight monthly evaluations until death in January 2013 due to hypovolemic shock following a dental extraction.

### Patient 2

A 15-month-old female infant from Georgia arrived in France in September 2010. She had fever, sweats, hepatosplenomegaly, pancytopenia and 213,000 *Leishmania*/ml. *Leishmania* amastigotes were observed in the bone marrow. There was no underlying immunosuppression. Treatment with 20 mg/kg of L-AmB resulted in complete resolution of symptoms and parasite clearance from the blood (PCR). Four months later, clinical symptoms recurred with 50 *Leishmania*/ml. The patient was treated with L-AmB then miltefosine for 28 days, resulting in complete clinical and parasitological response (Fig 1B). Five months later a second overt relapse occurred with typical symptoms and 20 *Leishmania*/ml. The patient was treated thereafter with meglumine antimoniate with a one-week break for QT interval prolongation. Upon EKG normalization, the course was reinstated with no further QT modifications. All VL clinical signs and laboratory abnormalities resolved. The patient remained relapse-free and PCR-negative 42 months of follow-up.

### Patient 3

A 37-year-old male from Georgia came to France in October 2012. He had HIV infection since 2006 inconstantly treated with HAART. Other medical conditions were adrenal insufficiency, cirrhosis due to chronic HCV, and treatment-controlled tuberculosis. VL was diagnosed in 2010 and treated in Georgia with meglumine antimoniate and L-AmB (doses and schedules unknown). In November 2012, he presented hepatosplenomegaly, lymphadenopathies, infiltrated cutaneous lesions, anemia, number of CD4 lymphocytes = 133/mm3, viral load < 50 copies/mL, and parasitemia of 6 *Leishmania*/ml. He received a course of L-AmB followed by maintenance therapy for 5 months (Fig 1B). In May 2013, he had weight loss, hepatosplenomegaly, anemia, number of CD4 lymphocytes = 177/mm3, viral load < 40 copies/mL and parasitemia of 12 *Leishmania*/ml. L-AmB treatment was intensified, but renal insufficiency and acute thrombocytopenia occurred. In June, the splenomegaly and parasitemia remained unchanged. A course of meglumine antimoniate was administered. Despite pre-existing liver disorders, the treatment was well tolerated except for mild neutropenia. One month later, a marked clinical improvement was observed and parasitemia decreased to 0.2 *Leishmania*/ml. In July, maintenance treatment with miltefosine (150 mg/day for 3 months) was started. Parasitemia was negative, the number of CD4 lymphocytes increased from 169 to 540/mm^3^, and viral load was undetectable. Parasitemia remained negative through 18 months.

### Patient 4

A 75-year-old male from Algeria had HIV infection since 2001 treated with nevirapine and lamivudine/zidovudine. He had cerebral toxoplasmosis, chronic hepatitis B, gastrointestinal CMV infection, and anal squamous cell carcinoma. In 2013, the patient started treatment with darunavir, ritonavir, raltegravir and emtricitabine. VL had been diagnosed in 2001, and treated with L-AmB 5 mg/Kg/d for 15 days with partial improvement. Maintenance therapy of 3 mg/kg weekly was planned but irregularly administered. Recurrence was reported in March 2011, the infecting specie was identified as *L*. *infantum*. The number of CD4 lymphocytes was 33/mm3, and viral load 75000 copies/mL. The patient received 40 mg/kg of L-AmB, followed by 3 mg/kg at 15-day interval. Clinical improvement was obtained but qualitative PCR remained positive. In 2012, L-AmB infusions were administered at 3-week intervals. In 2013, a last course of 50 mg/kg of L-AmB was administered, Despite this complete course of L-AmB, clinical symptoms worsened (weight loss, fatigue), clinical splenomegaly was present (greater diameter 20 cm on the CT scan), and parasitemia remained positive and stable (from 126 *Leishmania*/ml before treatment to 118 *Leishmania*/ml after completion of the course 3 weeks later). Meglumine antimoniate was thus administered, initially at low doses (4–10 mg/kg SbV every other day for 7 weeks) because of advanced age. Treatment was well tolerated except for moderate neutropenia. Fatigue resolved and weight increased from 50 to 61 Kg. The spleen was no longer palpable after 2 months of treatment. Number of CD4 lymphocytes increased from 75 to 134/mm3. Parasitemia cleared and remained negative through 13 months of follow-up.

## Discussion

In 4 patients with VL and secondary unresponsiveness or relapse after L-AmB treatment, second-line pentavalent antimony provided a sustained clinical and parasitological cure. A kidney transplant recipient developed relapsing episodes and received four 28–40 mg/kg courses of L-AmB, followed in 2 instances by prolonged L-AmB maintenance regimens. These attempts provided neither complete parasite clearance nor complete symptom resolution between overt episodes. In contrast, meglumine antimoniate treatment provided a sustained cure in a few weeks. The sustained positive outcome in this case cannot be unambiguously attributed to pentavalent antimony because of the patient’s underlying unstable immunosuppression. Nevertheless, this experience suggested that antimony could be an option in patients responding poorly to L-AmB. An immunocompetent infant relapsed twice after initially positive responses to L-AmB or L-AmB plus miltefosine. Again, upon treatment with antimony, sustained complete response was obtained. In 2 patients co-infected with HIV and *L*. *infantum* and responding poorly to 2–3 courses of L-AmB despite undetectable viral loads, sustained cure was similarly obtained after a single course of antimony, followed in one case by oral miltefosine. Taken together, these observations in different age groups and underlying conditions, advocate for the use of antimony in VL patients responding inadequately to L-AmB. L-AmB remains the antileishmanial agent with the widest therapeutic window in VL and should be used first-line wherever affordable for initial episodes. However, our experience confirms that unresponsiveness or repeated relapses occur in a small proportion of L-AmB-treated VL patients, especially when immunosuppression is present [12]. Switching from L-AmB to pentavalent antimony may be life-saving in these cases. Patients in this report were most likely infected with *L*. *infantum*. Whether our observations also apply to *L*. *donovani* thus remains to be fully confirmed but previous reports suggest that relapse of VL is frequent both in Indian [13] an East African [14] foci of *L*. *donovani* when patients treated with AmBisome have underlying immunosuppression.

Despite adequate antiretroviral therapy and undetectable viremia, the 2 HIV-infected patients failed to recover immunity during L-AmB treatment. After antimony treatment however, *Leishmania* blood PCR became negative and numbers of CD4 lymphocytes increased. Thus, beyond controlling the signs and symptoms of VL, the appropriate treatment of *Leishmania* infection may also contribute to the prevention of HIV-related complications. Organ transplant recipients can develop or reactivate visceral or muco-cutaneous leishmaniasis while under immunosuppressive therapy [15] such as high-dose prednisone [16]. In children, pentavalent antimony is generally effective and reasonably safe when used as a first-line therapy [1] [17] (which further supports its use in rare, complex cases with multiple relapses after treatment with L-AmB). By contrast, in other contexts the safety of pentavalent antimony is suboptimal, like in 9 of 59 (16%) HIV-positive Ethiopian patients treated with sodium stibogluconate for VL who died from antimony-induced adverse events [14]. In comparison, in our study, several laboratory abnormalities were observed, but by adapting the daily dose or administration rhythm, we were able to treat patients with a complete initial course (equivalent to 20 mg SbV/kg/day for 28 days) without life-threatening adverse events.

The complex histories of our 4 patients strongly suggest that L-AmB failure in VL is due to factors other than conventional antimicrobial resistance. *Leishmania* amastigotes exposed *in vivo* to amphotericin B for many episodes in immunosuppressed patients appear to be no less susceptible to the drug *in vitro* than primary isolates [18]. Although a positive effect of antimony on cell-mediated immunity cannot be excluded, we favor an hypothesis where unresponsiveness in patients could be related to dormant parasites, the quiescence protecting them from the lethal effect of L-AmB through a mechanism different from conventional resistance. For currently unknown reasons, dormant/quiescent parasites may retain sensitivity to antimony. Larger studies are needed to determine the respective contributions of parasite quiescence to antileishmanial agents and underlying immunosuppression in the occurrence of suboptimal response to L-AmB.
